# Dietary sugar and soft drink consumption in relation to atopic disease in children: evidence from the Polish global asthma network study

**DOI:** 10.1186/s12890-026-04430-9

**Published:** 2026-06-18

**Authors:** Charles Prendergast, Agnieszka Jarosińska, Grzegorz Brożek, Justyna Gawlewicz-Pawlik, Joanna Lenart-Mendera, Kamil Barański

**Affiliations:** Department of Epidemiology, Faculty of Medical Sciences in Katowice, Medical University of Silesia, 40-751 Katowice, Poland

**Keywords:** Fructose, Atopy, Asthma, Eczema, Rhinitis, Diet

## Abstract

**Background:**

Dietary sugars, particularly sugar rich foods and beverages, have been implicated in the pathogenesis of allergic diseases, but epidemiological evidence in children remains inconsistent.

**Objective:**

To examine associations between dietary sugar intake from snacks and soft drinks and atopic outcomes across multiple disease definitions in a large pediatric population.

**Methods:**

A cross-sectional analysis was conducted using data from the Polish arm of the Global Asthma Network (GAN) study (*n* = 4,647; ages 6–15 years). Dietary exposures, including frequency of sugary snack and soft drink consumption, were assessed using standardized questionnaires. Outcomes were categorized as current symptoms (wheeze, rhinitis, rhinoconjunctivitis, eczema), lifetime (“ever”) outcomes, and doctor-diagnosed disease.

**Results:**

Overall, 546 participants (12.2%) reported low sugary snack consumption, 1,827 (40.7%) consumed sugary snacks once or twice per week, and 2,113 (47.1%) reported consumption most or all days. For soft drinks, 1,889 participants (41.8%) reported low intake, 1,661 (36.8%) moderate intake, and 969 (21.4%) high intake. Sugary snack intake was not associated with current symptom-based outcomes. However, high sugary snack consumption was associated with increased odds of lifetime hay fever (aOR 1.42, 95% CI 1.12–1.80) and eczema (aOR 1.72, 95% CI 1.20–2.46), with weaker and inconsistent associations for doctor-diagnosed outcomes. In contrast, soft drink intake was inversely associated with allergic outcomes, including hay fever and eczema, but was positively associated with current wheeze (aOR 1.48, 95% CI 1.08–2.02). Female sex and traffic exposure were consistently associated with higher odds of current symptoms.

**Conclusions:**

Dietary sugar exposures are differentially associated with pediatric atopic disease depending on outcome definition. Positive associations with lifetime outcomes, alongside null findings for current symptoms and inverse associations for soft drinks, suggest a complex interplay of biological and behavioural factors, including reverse causation. Longitudinal and biomarker-based studies are needed to clarify causal pathways.

**Supplementary Information:**

The online version contains supplementary material available at 10.1186/s12890-026-04430-9.

## Introduction

Atopic diseases, including asthma, allergic rhinitis, and eczema, represent some of the most prevalent chronic conditions in children, with rising global prevalence and notable regional variation [1–3]. Epidemiological data indicate a sustained increase in childhood allergic conditions, consistent with broader European trends [4, 5]. Asthma remains the most prevalent pediatric chronic disease, while cases of eczema and rhinitis continue to rise globally [4–6].

Although genetic predisposition and environmental exposures are well-established contributors to atopic disease, emerging evidence highlights the significant impact of modifiable lifestyle factors, particularly dietary patterns, on the developing immune system [7]. Recent meta-analyses report a 28% increased risk of asthma associated with sugar-sweetened beverage consumption in children, and high free fructose intake was linked to a 2.7-fold elevated risk of asthma in pediatric populations (95% CI: 1.30–5.73) [8, 9].

In Poland, sucrose-derived energy intake, primarily from sweetened snacks, fizzy drinks and fast food exceeds WHO recommendations by nearly twofold among 5–6-year-old children [8]. More than 50% of Polish children in this age group consume sweets more than twice daily, reflecting a concerning trend of excessive sugar intake [8, 9]. This shift parallels the broader “Westernization” of the Polish diet, characterized by increased consumption of refined carbohydrates through sugary snacks, fast food, and sugar-sweetened beverages [10].

While genetic predisposition lays the foundation for atopy, dietary exposures act as potent environmental modifiers of allergic risk, particularly in early life [11, 12]. Diets high in sugars and processed fats, hallmarks of the Western diet, are consistently associated with increased prevalence of immune-mediated diseases, including atopic conditions [7, 11–14]. Polish pediatric studies show that children with respiratory allergies consume more fast food and sweetened beverages than healthy peers; these foods are typically high in fructose and low in essential micronutrients important for immune regulation 15. Regional studies suggest that urban dietary shifts, especially in post-industrial areas, may significantly influence allergic outcomes in Polish children [12, 13].

Fructose-containing sugars are common components of sugar-sweetened beverages, confectionery, and other foods characteristic of Western dietary patterns. Under conditions of high intake and caloric excess, fructose metabolism has been linked to lipogenesis, insulin resistance, oxidative stress, and inflammatory signalling [16, 17]. Experimental and epidemiological studies further suggest that sugar-rich and ultra-processed dietary patterns may influence allergic disease through effects on immune regulation, epithelial barrier function, and the gut microbiome [11, 13–22].

Despite strong mechanistic plausibility, population-level evidence linking dietary sugar exposure to pediatric atopy remains limited, particularly in Central and Eastern Europe. The present study aimed to quantify these associations within the Polish Global Asthma Network, hypothesizing that frequent consumption of sugar-rich food and beverages would be positively associated with atopic outcomes, potentially via immunometabolic mechanisms.

## Materials and methods

### Study design and participants

This cross-sectional analysis utilized data from the Polish arm of the Global Asthma Network (GAN) study conducted in the Katowice region. The GAN is an international collaborative initiative investigating the epidemiology of asthma and allergic diseases in children and adolescents. The present study included two pediatric cohorts: children aged 6–8 years and adolescents aged 13–15 years from the Katowice region.

### Data collection and instruments

Data were collected in the Silesia region of Poland using the standardized Phase I questionnaire of the Global Asthma Network (GAN), which follows and expands the validated International Study of Asthma and Allergies in Childhood (ISAAC) written instruments used in ISAAC Phase Three. The GAN methodology and questionnaire design have been previously published and described in detail [23] and build directly on the ISAAC core questionnaires [24].

The questionnaires were self-administered, completed by parents or legal guardians for the younger cohort (6–8 years) and by adolescents themselves for the older cohort (13–15 years). Items captured respiratory and allergic health, demographic characteristics, anthropometry (weight and height reported by legal guardians), and dietary habits over the preceding 12 months.

### Outcome definitions

Atopic outcomes were defined according to standardized GAN/ISAAC criteria and grouped into three domains: current symptoms during the past 12 months (wheeze, rhinitis, rhinoconjunctivitis, and eczema), lifetime (“ever”) outcomes (asthma ever, hay fever ever, and eczema ever), and doctor-diagnosed outcomes (asthma, hay fever, and eczema confirmed by a physician). All outcomes were coded as binary variables (1 = present, 0 = absent).

### Exposure variables

Although fructose-related mechanisms informed the biological rationale for this study, the exposures assessed in the present analysis were frequency-based measures of sugary snack and soft drink consumption rather than direct measures of fructose intake. Dietary exposures over the preceding 12 months were assessed using frequency-based questions. Sugar intake was defined as consumption of sweets/candies and soft drink intake as consumption of sugary beverages. Both exposures were recoded into three categories: low (never/occasionally), moderate (1–2 times/week), and high (most/all days).

These categories were defined a priori based on the distribution of responses in the dataset, interpretability, and consistency with commonly used frequency groupings in pediatric nutritional epidemiology. The use of categorical exposure levels was intended to facilitate interpretation of potential dose–response patterns while avoiding data-driven categorization.

### Covariates

Potential confounders, selected based on prior literature, included age (continuous, years), sex (male/female), body mass index (calculated from self-reported height/weight, analyzed continuously), physical activity (low/moderate/high), and traffic exposure near residence (never / seldom / frequent / almost all day). All categorical variables were recoded pre-analysis to ensure consistent reference categories.

### Statistical analysis

Population characteristics were summarized using frequencies and percentages for categorical variables and means ± standard deviation (SD) or medians with interquartile range (IQR) for continuous variables. Prevalence estimates were calculated for each atopic outcome. Associations between dietary exposures and atopic outcomes were examined using Pearson’s χ² tests, with cross-tabulations for sugar intake and soft drink consumption versus each outcome.

Potential effect modification was assessed by including interaction terms in multivariable models. Specifically, interactions between sugar intake and soft drink consumption, as well as between each dietary exposure and age and sex, were tested.

To account for multiple exposure–outcome testing, Benjamini–Hochberg false-discovery-rate correction was applied to the primary dietary exposure coefficients. The correction included 40 comparisons: moderate and high sugary snack intake and moderate and high soft drink intake across current, lifetime, and doctor-diagnosed atopic outcomes. Findings were interpreted using both nominal p-values and FDR-adjusted q-values. For statistically significant interaction terms, subgroup-specific adjusted odds ratios and 95% confidence intervals were estimated within the relevant strata to describe the direction and magnitude of effect modification.

Multivariable logistic regression models were used to estimate associations between dietary exposures (sugar intake and soft drink consumption) and atopic outcomes, with results presented as adjusted odds ratios (aORs) and 95% confidence intervals (CIs). All models were adjusted for age, sex, body mass index (BMI), physical activity, and traffic exposure. Additional analyses were conducted for doctor-diagnosed and lifetime outcomes using the same modeling approach. Statistical significance was set at *p* < 0.05. All analyses were performed using TIBCO Statistica software, version 13 (TIBCO Software Inc., Palo Alto, CA, USA).

### Handling of missing data

Multivariable analyses were conducted using complete-case analysis. The final analytic sample for regression models included 4,146 participants with complete data on exposures, outcomes, and covariates.

## Results

### Participant characteristics

A total of 4,647 participants were included in the analysis. The study population was evenly distributed by sex, comprising 2,339 males (50.3%) and 2,308 females (49.7%). The mean age was 11.5 ± 3.16 years (range 5–18). Among participants with available anthropometric data, the mean BMI was 18.3 ± 4.45, with a median of 18.3 (IQR 16.0–20.7). In terms of dietary exposures, 546 participants (12.2%) reported low sugary snack consumption, 1,827 (40.7%) consumed sugary snacks once or twice per week, and 2,113 (47.1%) reported consumption most or all days. For soft drinks, 1,889 participants (41.8%) reported low intake, 1,661 (36.8%) moderate intake, and 969 (21.4%) high intake. Regarding physical activity, 1,051 participants (23.1%) reported low activity levels, 1,477 (32.4%) moderate activity, and 2,026 (44.5%) high activity. Exposure to traffic-related air pollution varied, with 1,045 participants (22.9%) reporting low exposure, 2,068 (45.3%) moderate exposure, 923 (20.2%) high exposure, and 533 (11.7%) very high exposure to traffic near their residence. Descriptive analyses were based on the full study population (*N* = 4,647), whereas multivariable regression models were restricted to complete cases (*N* = 4,146).

### Prevalence of atopic outcomes

The prevalence of atopic outcomes in the study population is presented in Table 1. Among current (12-month) symptoms, rhinitis was the most commonly reported condition, affecting 1,115 participants (24.0%), followed by eczema in 650 participants (14.0%), rhinoconjunctivitis in 534 (11.5%), and wheeze in 435 (9.4%).

**Table 1:** Prevalence of atopic outcomes in the study population (*N* = 4,647)

Outcome	Cases *n* (%)
Current symptoms (past 12 months)	
Wheeze	435 (9.4)
Rhinitis	1,115 (24.0)
Rhinoconjunctivitis	534 (11.5)
Eczema	650 (14.0)
Lifetime (“ever”) outcomes	
Asthma (ever)	420 (9.0)
Hay fever (ever)	1,228 (26.4)
Eczema (ever)	564 (12.1)
Doctor-diagnosed outcomes	
Asthma (doctor-diagnosed)	387 (8.3)
Hay fever (doctor-diagnosed)	863 (18.6)
Eczema (doctor-diagnosed)	411 (8.8)

For lifetime (“ever”) outcomes, hay fever was the most prevalent condition, reported in 1,228 participants (26.4%), while eczema and asthma were reported in 564 (12.1%) and 420 (9.0%) participants, respectively.

Doctor-diagnosed outcomes showed a similar pattern, with hay fever again being the most common (863 cases, 18.6%), followed by eczema (411 cases, 8.8%) and asthma (387 cases, 8.3%).

Overall, allergic conditions affecting the upper airways (rhinitis and hay fever) were more prevalent than asthma across all outcome definitions, while eczema demonstrated intermediate prevalence levels.

### Multivariable associations with current atopic symptoms

Multivariable associations between dietary exposures and current (12-month) atopic symptoms are presented in Table 2.

**Table 2:** Associations between dietary exposures and current (12-month) atopic symptoms (*N* = 4,146)

Outcome	Cases *n* (%)	Predictor	Comparison	aOR	95% CI
Current wheeze	390 (9.4)	Sugar intake	Moderate vs. low	0.94	0.66–1.34
			High vs. low	1.08	0.76–1.53
		Soft drink intake	Moderate vs. low	1.26	0.96–1.65
			High vs. low	**1.48**	**1.08–2.02**
		Age (per year)	—	1.04	1.00–1.09
		BMI (per unit)	—	**1.02**	**1.00–1.03**
		Sex	Female vs. male	**1.31**	**1.06–1.62**
		Exercise	1–2×/week vs. low	1.16	0.86–1.57
			≥ 3×/week vs. low	1.13	0.85–1.51
		Traffic	Seldom vs. never	0.93	0.70–1.23
			Frequent vs. never	1.02	0.74–1.42
			Almost all day vs. never	**1.65**	**1.16–2.33**
Current eczema	591 (14.3)	Sugar intake	Moderate vs. low	1.00	0.74–1.35
			High vs. low	1.14	0.85–1.53
		Soft drink intake	Moderate vs. low	**0.79**	**0.64–0.99**
			High vs. low	0.92	0.70–1.20
		Age (per year)	—	**0.96**	**0.93–0.99**
		BMI (per unit)	—	1.01	0.99–1.02
		Sex	Female vs. male	**1.85**	**1.54–2.22**
		Exercise	1–2×/week vs. low	**1.38**	**1.07–1.77**
			≥ 3×/week vs. low	**1.39**	**1.09–1.78**
		Traffic	Seldom vs. never	0.91	0.72–1.15
			Frequent vs. never	1.10	0.84–1.44
			Almost all day vs. never	**1.47**	**1.09–1.99**
Current rhinitis	1,039 (25.1)	Sugar intake	Moderate vs. low	1.23	0.97–1.57
			High vs. low	1.18	0.93–1.51
		Soft drink intake	Moderate vs. low	**0.73**	**0.61–0.87**
			High vs. low	**0.65**	**0.52–0.81**
		Age (per year)	—	**1.03**	**1.00–1.06**
		BMI (per unit)	—	**1.01**	**1.00–1.02**
		Sex	Female vs. male	**1.24**	**1.07–1.44**
		Exercise	1–2×/week vs. low	**1.25**	**1.02–1.52**
			≥ 3×/week vs. low	1.19	0.98–1.44
		Traffic	Seldom vs. never	0.87	0.72–1.05
			Frequent vs. never	1.10	0.88–1.36
			Almost all day vs. never	**1.48**	**1.15–1.89**
Current rhinoconjunctivitis	502 (12.1)	Sugar intake	Moderate vs. low	1.22	0.88–1.69
			High vs. low	1.35	0.97–1.87
		Soft drink intake	Moderate vs. low	0.81	0.64–1.02
			High vs. low	0.76	0.57–1.01
		Age (per year)	—	**1.11**	**1.06–1.15**
		BMI (per unit)	—	1.01	1.00–1.03
		Sex	Female vs. male	**1.60**	**1.31–1.95**
		Exercise	1–2×/week vs. low	**1.61**	**1.20–2.16**
			≥ 3×/week vs. low	**1.65**	**1.24–2.18**
		Traffic	Seldom vs. never	0.91	0.71–1.19
			Frequent vs. never	1.31	0.98–1.75
			Almost all day vs. never	**1.52**	**1.09–2.11**

Adjusted for age, sex, BMI, physical activity, traffic exposure, sugar intake, and soft drink intake. Reference categories: sugar and soft drink intake = never or only occasionally; exercise = never or occasionally; traffic = never; sex = male. Age and BMI modeled as continuous variables. Statistically significant associations (*p* < 0.05) are shown in bold

### Sugar intake

No statistically significant associations were observed between sugar intake and any current atopic symptoms. Odds ratios for both moderate and high sugar consumption were close to unity across all outcomes, including wheeze, eczema, rhinitis, and rhinoconjunctivitis, indicating no consistent relationship between sugar intake and current symptom burden.

### Soft drink consumption

Soft drink consumption showed heterogeneous associations across outcomes. High soft drink intake was associated with increased odds of current wheeze (aOR 1.48, 95% CI 1.08–2.02), while moderate intake showed a similar but non-significant trend.

In contrast, soft drink intake was inversely associated with allergic symptoms. Moderate consumption was associated with lower odds of current eczema (aOR 0.79, 95% CI 0.64–0.99), and both moderate and high intake were associated with reduced odds of current rhinitis (aOR 0.73, 95% CI 0.61–0.87 and aOR 0.65, 95% CI 0.52–0.81, respectively). Similar inverse trends were observed for rhinoconjunctivitis, although these did not reach statistical significance.

### Covariates

Several covariates demonstrated consistent associations with current atopic symptoms.

Female sex was associated with higher odds of all current outcomes, including wheeze (aOR 1.31, 95% CI 1.06–1.62), eczema (aOR 1.85, 95% CI 1.54–2.22), rhinitis (aOR 1.24, 95% CI 1.07–1.44), and rhinoconjunctivitis (aOR 1.60, 95% CI 1.31–1.95).

Physical activity was positively associated with several outcomes. Moderate levels of activity were associated with increased odds of eczema (aOR 1.38, 95% CI 1.07–1.77), rhinitis (aOR 1.25, 95% CI 1.02–1.52), and rhinoconjunctivitis (aOR 1.61, 95% CI 1.20–2.16), while high activity levels were associated with eczema (aOR 1.39, 95% CI 1.09–1.78) and rhinoconjunctivitis (aOR 1.65, 95% CI 1.24–2.18).

Exposure to traffic showed a consistent positive association with symptom outcomes. Living in areas with traffic exposure “almost all day” was associated with higher odds of wheeze (aOR 1.65, 95% CI 1.16–2.33), eczema (aOR 1.47, 95% CI 1.09–1.99), rhinitis (aOR 1.48, 95% CI 1.15–1.89), and rhinoconjunctivitis (aOR 1.52, 95% CI 1.09–2.11).

Age showed divergent associations, being positively associated with wheeze, rhinitis, and rhinoconjunctivitis, but inversely associated with eczema (aOR 0.96, 95% CI 0.93–0.99). Higher BMI was associated with a small increase in the odds of wheeze and rhinitis.

### Multivariable associations with doctor-diagnosed atopic outcomes

Multivariable associations between dietary exposures and doctor-diagnosed atopic outcomes are presented in Table 3.

**Table 3:** Associations between dietary exposures and doctor-diagnosed atopic outcomes (*N* = 4,146)

Outcome	Cases *n* (%)	Predictor	Comparison	aOR	95% CI
Asthma (doctor-diagnosed)	345 (8.3)	Sugar intake	Moderate vs. low	0.98	0.69–1.40
			High vs. low	0.84	0.58–1.20
		Soft drink intake	Moderate vs. low	1.18	0.89–1.55
			High vs. low	1.09	0.77–1.53
		Age	Per year	1.03	0.99–1.08
		BMI	Per unit	1.00	0.98–1.01
		Sex	Female vs. male	0.77	0.62–0.97
		Exercise	1–2×/week vs. low	0.94	0.70–1.28
			≥ 3×/week vs. low	0.93	0.69–1.24
		Traffic	Seldom vs. never	0.83	0.63–1.11
			Frequent vs. never	0.96	0.69–1.34
			Almost all day vs. never	1.21	0.83–1.76
Eczema (doctor-diagnosed)	378 (9.1)	Sugar intake	Moderate vs. low	1.09	0.72–1.65
			High vs. low	1.48	0.99–2.22
		Soft drink intake	Moderate vs. low	0.77	0.59–1.02
			High vs. low	0.65	0.43–0.98
		Age	Per year	0.79	0.76–0.82
		BMI	Per unit	1.01	0.99–1.03
		Sex	Female vs. male	1.23	0.98–1.53
		Exercise	1–2×/week vs. low	1.52	1.13–2.04
			≥ 3×/week vs. low	1.32	0.98–1.79
		Traffic	Seldom vs. never	0.81	0.61–1.07
			Frequent vs. never	1.03	0.75–1.43
			Almost all day vs. never	1.42	0.98–2.05
Hay fever (doctor-diagnosed)	785 (18.9)	Sugar intake	Moderate vs. low	1.05	0.80–1.37
			High vs. low	1.29	0.99–1.69
		Soft drink intake	Moderate vs. low	0.76	0.62–0.92
			High vs. low	0.71	0.55–0.90
		Age	Per year	0.98	0.95–1.01
		BMI	Per unit	1.01	1.00–1.02
		Sex	Female vs. male	0.84	0.72–0.99
		Exercise	1–2×/week vs. low	1.11	0.89–1.38
			≥ 3×/week vs. low	1.03	0.84–1.27
		Traffic	Seldom vs. never	0.86	0.70–1.05
			Frequent vs. never	1.09	0.86–1.37
			Almost all day vs. never	1.03	0.78–1.36

Adjusted for age, sex, BMI, physical activity, traffic exposure, sugar intake, and soft drink intake. Reference categories: sugar and soft drink intake = never or only occasionally; exercise = never or occasionally; traffic = never; sex = male. Age and BMI modeled as continuous variables

### Sugar intake

Sugar intake was not significantly associated with doctor-diagnosed asthma or hay fever. For eczema, higher sugar intake showed a positive but borderline association, with increased odds observed for high versus low consumption (aOR 1.48, 95% CI 0.99–2.22), although this did not reach statistical significance.

### Soft drink consumption

Soft drink consumption demonstrated inverse associations with allergic outcomes. High soft drink intake was associated with lower odds of doctor-diagnosed eczema (aOR 0.65, 95% CI 0.43–0.98). Similarly, both moderate and high soft drink intake were associated with reduced odds of doctor-diagnosed hay fever (aOR 0.76, 95% CI 0.62–0.92 and aOR 0.71, 95% CI 0.55–0.90, respectively).

No significant associations were observed between soft drink consumption and doctor-diagnosed asthma.

### Covariates

Female sex was associated with lower odds of doctor-diagnosed asthma (aOR 0.77, 95% CI 0.62–0.97) and hay fever (aOR 0.84, 95% CI 0.72–0.99), while no significant association was observed for eczema.

Age was inversely associated with doctor-diagnosed eczema (aOR 0.79, 95% CI 0.76–0.82), indicating lower odds with increasing age. BMI was not meaningfully associated with any doctor-diagnosed outcomes.

Physical activity was positively associated with eczema, with moderate activity levels associated with increased odds (aOR 1.52, 95% CI 1.13–2.04), while no consistent associations were observed for asthma or hay fever.

Traffic exposure was not significantly associated with doctor-diagnosed outcomes.

### Multivariable associations with lifetime (“Ever”) atopic outcomes

Multivariable associations between dietary exposures and lifetime (“ever”) atopic outcomes are presented in Table 4.

**Table 4:** Associations between dietary exposures and lifetime (“ever”) atopic outcomes (*N* = 4,146)

Outcome	Cases *n* (%)	Predictor	Comparison	aOR	95% CI
Asthma (ever)	376 (9.1)	Sugar intake	Moderate vs. low	0.91	0.65–1.29
			High vs. low	0.85	0.60–1.20
		Soft drink intake	Moderate vs. low	1.18	0.90–1.53
			High vs. low	1.06	0.77–1.48
		Age (per year)	—	1.04	1.00–1.08
		BMI (per unit)	—	1.00	0.98–1.01
		Sex	Female vs. male	0.81	0.65–1.01
		Exercise	1–2×/week vs. low	1.11	0.83–1.50
			≥ 3×/week vs. low	1.01	0.76–1.34
		Traffic	Seldom vs. never	0.81	0.62–1.07
			Frequent vs. never	0.91	0.66–1.26
			Almost all day vs. never	1.15	0.80–1.65
Hay fever (ever)	1,120 (27.0)	Sugar intake	Moderate vs. low	1.07	0.85–1.36
			High vs. low	**1.42**	**1.12–1.80**
		Soft drink intake	Moderate vs. low	**0.79**	**0.66–0.93**
			High vs. low	**0.71**	**0.57–0.87**
		Age (per year)	—	**1.04**	**1.01–1.07**
		BMI (per unit)	—	**1.01**	**1.00–1.02**
		Sex	Female vs. male	1.09	0.95–1.25
		Exercise	1–2×/week vs. low	1.10	0.91–1.33
			≥ 3×/week vs. low	1.03	0.85–1.24
		Traffic	Seldom vs. never	0.96	0.80–1.15
			Frequent vs. never	1.12	0.91–1.38
			Almost all day vs. never	1.12	0.88–1.44
Eczema (ever)	521 (12.6)	Sugar intake	Moderate vs. low	1.29	0.89–1.85
			High vs. low	**1.72**	**1.20–2.46**
		Soft drink intake	Moderate vs. low	**0.78**	**0.62–0.99**
			High vs. low	**0.71**	**0.52–0.99**
		Age (per year)	—	**0.84**	**0.81–0.87**
		BMI (per unit)	—	1.01	0.99–1.03
		Sex	Female vs. male	**1.24**	**1.02–1.50**
		Exercise	1–2×/week vs. low	**1.50**	**1.16–1.93**
			≥ 3×/week vs. low	1.24	0.96–1.61
		Traffic	Seldom vs. never	0.87	0.68–1.11
			Frequent vs. never	1.07	0.80–1.42
			Almost all day vs. never	**1.43**	**1.03–1.97**

Adjusted for age, sex, BMI, physical activity, traffic exposure, sugar intake, and soft drink intake. Reference categories: sugar and soft drink intake = never or only occasionally; exercise = never or occasionally; traffic = never; sex = male. Age and BMI modeled as continuous variables. Statistically significant associations (*p* < 0.05) are shown in bold

### Sugar intake

Higher sugar intake was associated with increased odds of allergic outcomes. Children with high sugar consumption had significantly higher odds of hay fever (aOR 1.42, 95% CI 1.12–1.80) and eczema (aOR 1.72, 95% CI 1.20–2.46) compared with those with low intake. No statistically significant association was observed for moderate sugar intake, although effect estimates were directionally positive.

No association was observed between sugar intake and asthma ever.

#### Soft drink consumption

Soft drink intake was inversely associated with allergic outcomes. Both moderate and high soft drink consumption were associated with reduced odds of hay fever (aOR 0.79, 95% CI 0.66–0.93 and aOR 0.71, 95% CI 0.57–0.87, respectively). A similar pattern was observed for eczema, where both moderate and high intake were associated with lower odds (aOR 0.78, 95% CI 0.62–0.99 and aOR 0.71, 95% CI 0.52–0.99, respectively).

No significant associations were observed between soft drink intake and asthma ever.

### Covariates

Age was positively associated with hay fever (aOR 1.04, 95% CI 1.01–1.07) and asthma ever (aOR 1.04, 95% CI 1.00–1.08), but inversely associated with eczema (aOR 0.84, 95% CI 0.81–0.87). BMI showed a small positive association with hay fever.

Female sex was associated with increased odds of eczema (aOR 1.24, 95% CI 1.02–1.50), while no significant sex differences were observed for asthma or hay fever.

Physical activity was positively associated with eczema, with moderate activity levels associated with higher odds (aOR 1.50, 95% CI 1.16–1.93). No consistent associations were observed for asthma or hay fever.

High levels of traffic exposure were associated with increased odds of eczema (aOR 1.43, 95% CI 1.03–1.97), while no clear associations were observed for asthma or hay fever.

#### Effect modification and age-stratified analyses

We assessed potential effect modification by sex and age group in multivariable models. A significant interaction between sugary snack intake and sex was observed for lifetime hay fever (Wald χ² = 13.22, *p* = 0.0013), indicating that the association differed between males and females. Among males, both moderate sugary snack intake (aOR 1.66, 95% CI 1.12–2.45) and high sugary snack intake (aOR 2.24, 95% CI 1.52–3.32) were associated with higher odds of lifetime hay fever. In contrast, no clear association was observed among females for either moderate intake (aOR 0.81, 95% CI 0.59–1.10) or high intake (aOR 1.02, 95% CI 0.76–1.39).

Evidence of effect modification by age group was also observed for the association between sugary snack intake and lifetime eczema (Wald χ² = 8.18, *p* = 0.0167). In the younger parent-reported cohort, both moderate intake (aOR 2.25, 95% CI 1.21–4.19) and high intake (aOR 2.58, 95% CI 1.40–4.75) were associated with higher odds of lifetime eczema. In the older self-reported adolescent cohort, estimates were weaker and not statistically significant for moderate intake (aOR 0.92, 95% CI 0.59–1.45) or high intake (aOR 1.30, 95% CI 0.83–2.02). Other interaction terms were not statistically significant. These subgroup estimates are presented in Supplementary Table 3.

In additional models including age group as a categorical predictor, the main dietary exposure estimates were similar to the primary analyses. However, age group was associated with several outcomes: compared with the younger parent-reported cohort, the older self-reported cohort had higher odds of lifetime hay fever and lower odds of current and lifetime eczema, supporting cautious interpretation of pooled analyses (Supplementary Table 1).

Age-stratified analyses suggested that heterogeneity was concentrated in eczema-related outcomes. In the younger parent-reported cohort, sugary snack intake was positively associated with current eczema, lifetime eczema, and doctor-diagnosed eczema, whereas corresponding estimates in the older self-reported cohort were weaker and generally not statistically significant. The strongest and most consistent pattern was observed for lifetime eczema, where both moderate intake (aOR 2.25, 95% CI 1.21–4.19) and high intake (aOR 2.58, 95% CI 1.40–4.75) were associated with higher odds in the younger cohort, compared with weaker estimates in the older cohort. For non-eczema outcomes, stratified associations were less consistent and often imprecise. High soft drink intake was positively associated with current wheeze in the older cohort but not in the younger cohort, whereas inverse associations between soft drink intake and current rhinitis were observed in both cohorts. Full age-stratified estimates are presented in Supplementary Table 4.

#### False discovery rate correction

After false-discovery-rate correction, the positive associations between high sugary snack intake and lifetime hay fever and lifetime eczema remained statistically significant. Inverse associations between soft drink intake and current rhinitis, doctor-diagnosed hay fever, and lifetime hay fever also remained significant. However, several nominal associations, including the positive association between high soft drink intake and current wheeze, did not remain significant after correction (Supplementary Table 2).

## Discussion

This study provides population-based evidence from the Polish Global Asthma Network (GAN) cohort on associations between dietary sugar exposures and atopic disease across three outcome domains: current symptoms, lifetime (“ever”) outcomes, and doctor-diagnosed disease. A consistent pattern emerged. While no clear associations were observed for current symptoms, higher sugar intake was positively associated with lifetime allergic disease, particularly hay fever and eczema, whereas soft drink consumption showed inverse associations across multiple outcomes. We additionally assessed effect modification by sex and age group. A significant interaction between sugar intake and sex was observed for lifetime hay fever, indicating that the association differed between boys and girls, possibly reflecting behavioural, reporting, or biological differences. Heterogeneity by age group was identified for the association between sugar intake and lifetime eczema. Given differences in exposure assessment (parent-reported vs. self-reported), this may reflect either true age-related variation or measurement differences. Most other interactions were not statistically significant, suggesting overall consistency across subgroups.

### Synthesis of findings

A key finding is the divergence between outcome domains. Sugary snack intake was not associated with current symptoms but showed positive associations with lifetime allergic outcomes, particularly hay fever and eczema. Associations with doctor-diagnosed outcomes were weaker. This pattern may reflect differences in reporting, recall, diagnosis, or behavioural modification rather than acute symptom burden. Soft drink consumption showed an opposing pattern, with inverse associations for lifetime and doctor-diagnosed outcomes and mixed associations for current symptoms, including a positive association with wheeze. The inverse associations for soft drinks should not be interpreted as protective and may reflect reverse causation, residual confounding, or exposure misclassification. These findings indicate that different exposure items may reflect different dietary behaviours or reporting patterns, although this was not empirically tested using dietary pattern analysis. Additionally, the opposing directions of association observed for sugary snacks and soft drinks suggest that these exposure variables may be capturing different behavioural or socioeconomic constructs rather than reflecting the biological effects of sugar exposure alone.

### Exposure–outcome complexity

The contrasting associations between sugary foods and beverages represent a central finding. Sugary snacks, reflecting confectionery and ultra-processed foods, were positively associated with allergic outcomes, whereas soft drinks were inversely associated with hay fever and eczema. In principle, sugar-sweetened beverages may contribute to rapidly absorbable sugar exposure, including fructose-containing sugars overwhelming hepatic fructose metabolic pathways leading to uric acid production, de novo lipogenesis and oxidative stress [14, 19, 20]; however, the present questionnaire did not quantify beverage composition, sugar dose, or fructose content. Therefore, mechanistic interpretations should remain tentative. Sugary snack intake may better capture broader dietary patterns, including ultra-processed food consumption linked to systemic inflammation and immune dysregulation [25]. These differences likely reflect combined metabolic, behavioural, and contextual factors. However, the inverse associations observed here argue against a straightforward mechanistic interpretation and instead suggest the influence of behavioural factors such as reverse causation or residual confounding.

### Interpretation and reverse causation

Inverse associations for soft drinks should be interpreted cautiously. Possible explanations include reverse causation, protopathic bias, residual confounding, exposure misclassification, differential reporting by age group, and chance findings arising from multiple testing [26, 27]. The discrepancy between current and lifetime outcomes supports this explanation, suggesting behavioural modification after diagnosis may attenuate associations in cross-sectional analyses. Additionally, frequency-based exposure assessment may introduce nondifferential misclassification, likely biasing estimates toward the null and potentially underestimating true associations.

### Covariate patterns and environmental signals

Several covariates showed consistent associations. Female sex was linked to higher odds of eczema, rhinitis, and rhinoconjunctivitis, but lower odds of asthma, reflecting known sex-related immune and hormonal influences. Physical activity was positively associated with some outcomes, possibly reflecting increased environmental exposure, though reverse causation and residual confounding cannot be excluded. Traffic exposure was consistently associated with current symptoms, particularly wheeze, rhinitis, and rhinoconjunctivitis, supporting existing evidence linking air pollution to allergic disease and reinforcing the internal validity of the dataset [26].

### Mechanistic context

The observed positive associations between sugar intake and allergic outcomes are broadly consistent with prior literature, although the mechanisms underlying these relationships cannot be directly assessed in the present study. While high sugar intake has been linked to metabolic and immune perturbations in experimental and clinical contexts [11, 14, 15], our exposure measures do not capture specific nutrient composition or quantity, and therefore any mechanistic interpretation should be considered speculative.

Previous studies suggest that high sugar intake and consequent fructose metabolism may increase uric acid production and oxidative stress [27–34], processes that have been associated with airway inflammation [35] and allergic conditions [36, 37]. Additionally, fructose exposure has been linked to pro-inflammatory cytokine production and Th2-skewed immune responses in experimental models [19], which could plausibly influence epithelial barrier function and sensitization pathways [38].

Similarly, hypotheses involving the gut–lung axis propose that high sugar intake may influence allergic disease through effects on the intestinal microbiome and barrier integrity [39, 40], potentially facilitating systemic inflammatory signalling via microbial products such as lipopolysaccharide [41, 42].

#### Strengths and limitations

This study has several strengths, including a large population-based sample, standardized outcome definitions based on GAN/ISAAC instruments, and the ability to examine multiple outcome domains within the same cohort, consistent with other studies from the region [43–45]. The use of consistent multivariable models across outcomes further enhances comparability and the multiple-comparison analysis indicates that some nominally significant associations should be interpreted cautiously. In particular, the association between high soft drink intake and current wheeze was attenuated after FDR correction. The findings that remained significant after correction were concentrated in lifetime hay fever and eczema for sugary snack intake and inverse associations for soft drink intake. These results support interpreting the study as exploratory and hypothesis-generating rather than confirmatory.

However, several limitations should be considered. The cross-sectional design precludes causal inference and is susceptible to reverse causation. Dietary exposures were assessed using self- or parent-reported frequency measures without quantitative intake data or biomarker validation, introducing potential measurement error. The study combined two age groups with different sources of reporting, with parents or guardians completing questionnaires for younger children and adolescents self-reporting in the older group; although most exploratory analyses did not indicate meaningful heterogeneity by age group, some differential measurement error cannot be excluded. Residual confounding by socioeconomic factors, parental atopy, or broader dietary patterns also cannot be excluded. Importantly, the potential direction of bias due to unmeasured confounding should be considered. Factors such as parental atopy, overall dietary patterns, and socioeconomic status are likely associated both with dietary behaviours and the risk of allergic disease. For example, children from higher socioeconomic backgrounds may have both healthier dietary patterns (including lower consumption of ultra-processed sugary snacks) and a higher likelihood of receiving a medical diagnosis, which could bias associations in opposite directions depending on the outcome definition [46]. Similarly, unmeasured health-conscious behaviours may lead to reduced sugar intake among children with pre-existing allergic conditions, potentially biasing estimates toward inverse associations, particularly for soft drink consumption [47–49]. Conversely, residual confounding by unhealthy lifestyle patterns may lead to overestimation of positive associations observed for sugary snacks. Taken together, these mechanisms suggest that the observed associations may reflect a combination of true biological effects and residual confounding acting in both directions and should therefore be interpreted with caution. Residual confounding remains an important limitation. Direct measures of parental atopy and socioeconomic status were not available in the analytic dataset and therefore could not be included in the multivariable models. These factors may be associated with both dietary behaviours and allergic disease risk, as well as with healthcare access and likelihood of receiving a doctor diagnosis. Consequently, the observed associations may be biased in either direction and should not be interpreted causally. In addition, doctor-diagnosed outcomes may reflect healthcare access and diagnostic practices. Finally, multiple comparisons increase the risk of type I error, and the findings should therefore be interpreted as associative rather than causal.

## Conclusion

This study provides population-based evidence that dietary sugar exposures are differentially associated with pediatric atopic disease, with important variation across outcome definitions and sources of intake. Higher consumption of sugar-rich foods was associated with selected lifetime allergic outcomes, particularly hay fever and eczema, whereas no clear associations were observed for current symptom-based outcomes. In contrast, soft drink intake showed inverse associations with allergic disease, most plausibly reflecting behavioural modification or reverse causation in children with established conditions.

These findings highlight the complexity of dietary exposures in atopic disease epidemiology and underscore the importance of distinguishing between food sources, patterns of consumption, and outcome definitions. The observed divergence between current, lifetime, and doctor-diagnosed outcomes suggests that reliance on a single outcome measure may obscure meaningful exposure–disease relationships.

Future longitudinal studies incorporating objective dietary assessment and metabolic biomarkers are needed to clarify temporality, reduce exposure misclassification, and better characterize associations between dietary sugar exposures and atopic outcomes.

## Supplementary Information

Supplementary Material 1.

## Data Availability

The datasets generated and/or analysed during the current study are not publicly available but are available from the corresponding author on reasonable request and with permission of the Global Asthma Network.

## Declarations

Written informed consent was obtained from the parents or legal guardians of all participating children and adolescents prior to inclusion in the study.

## Abbreviations

GAN: Global asthma network
ISAAC: International study of asthma and allergies in childhood
aOR: Adjusted odds ratio
CI: Confidence interval
ROS: Reactive oxygen species
LPS: Lipopolysaccharide
FDR: False discovery rate
