# Supplementary material for: Dietary sugar and soft drink consumption in relation to atopic disease in children: evidence from the Polish global asthma network study
Source: BMC Pulm Med. 2026 Jun 18;26:320. doi: 10.1186/s12890-026-04430-9 (PMC13383431; doi:10.1186/s12890-026-04430-9)
Supplement: Supplementary file 1 — Supplementary Material 1. [file 12890_2026_4430_MOESM1_ESM.docx]

**Supplementary Table 1:** Age group and BMI associations across outcomes. Age Group 1, the younger parent-reported cohort, was the reference category; age-group odds ratios compare Age Group 2, the older self-reported adolescent cohort, with Age Group 1. Models were adjusted for sugary snack intake, soft drink intake, sex, BMI, physical activity, and traffic exposure. For sugary snack and soft drink intake, low intake was the reference category.

| **Outcome** | **Predictor** | **aOR** | **95% CI** |
| --- | --- | --- | --- |
| Current wheeze | Age group | 1.27 | 0.94–1.70 |
| Current wheeze | BMI | 1.02 | 1.00–1.04 |
| Current rhinitis | Age group | 1.16 | 0.97–1.40 |
| Current rhinitis | BMI | 1.01 | 1.00–1.02 |
| Current rhinoconjunctivitis | Age group | 1.85 | 1.42–2.41 |
| Current rhinoconjunctivitis | BMI | 1.01 | 1.00–1.03 |
| Current eczema | Age group | 0.74 | 0.59–0.92 |
| Current eczema | BMI | 1.01 | 0.99–1.02 |
| Asthma ever | Age group | 1.32 | 0.99–1.77 |
| Asthma ever | BMI | 1.00 | 0.98–1.01 |
| Hay fever ever | Age group | 1.26 | 1.05–1.51 |
| Hay fever ever | BMI | 1.01 | 1.00–1.02 |
| Eczema ever | Age group | 0.31 | 0.24–0.39 |
| Eczema ever | BMI | 1.01 | 1.00–1.03 |

**Supplementary Table 2**: Benjamini–Hochberg false-discovery-rate correction for dietary exposure associations.

| **Outcome** | **Exposure** | **Comparison** | **aOR** | **95% CI** | **p-value** | **FDR q-value** |
| --- | --- | --- | --- | --- | --- | --- |
| Current wheeze | Sugar intake | Moderate vs low | 0.94 | 0.66–1.34 | 0.731 | 0.771 |
| Current wheeze | Sugar intake | High vs low | 1.08 | 0.76–1.53 | 0.684 | 0.771 |
| Current wheeze | Soft drink intake | Moderate vs low | 1.26 | 0.96–1.65 | 0.090 | 0.180 |
| Current wheeze | Soft drink intake | High vs low | 1.48 | 1.08–2.02 | 0.015 | 0.067 |
| Current eczema | Sugar intake | Moderate vs low | 1.00 | 0.74–1.35 | 0.995 | 0.995 |
| Current eczema | Sugar intake | High vs low | 1.14 | 0.85–1.53 | 0.392 | 0.522 |
| Current eczema | Soft drink intake | Moderate vs low | 0.79 | 0.64–0.99 | 0.037 | 0.129 |
| Current eczema | Soft drink intake | High vs low | 0.92 | 0.70–1.20 | 0.542 | 0.722 |
| Current rhinitis | Sugar intake | Moderate vs low | 1.23 | 0.97–1.57 | 0.089 | 0.180 |
| Current rhinitis | Sugar intake | High vs low | 1.18 | 0.93–1.51 | 0.172 | 0.313 |
| Current rhinitis | Soft drink intake | Moderate vs low | 0.73 | 0.61–0.87 | <0.001 | 0.010 |
| Current rhinitis | Soft drink intake | High vs low | 0.65 | 0.52–0.81 | <0.001 | 0.010 |
| Current rhinoconjunctivitis | Sugar intake | Moderate vs low | 1.22 | 0.88–1.69 | 0.236 | 0.369 |
| Current rhinoconjunctivitis | Sugar intake | High vs low | 1.35 | 0.97–1.87 | 0.073 | 0.162 |
| Current rhinoconjunctivitis | Soft drink intake | Moderate vs low | 0.81 | 0.64–1.02 | 0.071 | 0.162 |
| Current rhinoconjunctivitis | Soft drink intake | High vs low | 0.76 | 0.57–1.01 | 0.057 | 0.148 |
| Doctor-diagnosed asthma | Sugar intake | Moderate vs low | 0.98 | 0.69–1.40 | 0.908 | 0.931 |
| Doctor-diagnosed asthma | Sugar intake | High vs low | 0.84 | 0.58–1.20 | 0.340 | 0.503 |
| Doctor-diagnosed asthma | Soft drink intake | Moderate vs low | 1.18 | 0.89–1.55 | 0.242 | 0.369 |
| Doctor-diagnosed asthma | Soft drink intake | High vs low | 1.09 | 0.77–1.53 | 0.638 | 0.771 |
| Doctor-diagnosed eczema | Sugar intake | Moderate vs low | 1.09 | 0.72–1.65 | 0.696 | 0.771 |
| Doctor-diagnosed eczema | Sugar intake | High vs low | 1.48 | 0.99–2.22 | 0.056 | 0.148 |
| Doctor-diagnosed eczema | Soft drink intake | Moderate vs low | 0.77 | 0.59–1.02 | 0.067 | 0.162 |
| Doctor-diagnosed eczema | Soft drink intake | High vs low | 0.65 | 0.43–0.98 | 0.041 | 0.129 |
| Doctor-diagnosed hay fever | Sugar intake | Moderate vs low | 1.05 | 0.80–1.37 | 0.744 | 0.771 |
| Doctor-diagnosed hay fever | Sugar intake | High vs low | 1.29 | 0.99–1.69 | 0.059 | 0.148 |
| Doctor-diagnosed hay fever | Soft drink intake | Moderate vs low | 0.76 | 0.62–0.92 | 0.005 | 0.029 |
| Doctor-diagnosed hay fever | Soft drink intake | High vs low | 0.71 | 0.55–0.90 | 0.005 | 0.029 |
| Asthma ever | Sugar intake | Moderate vs low | 0.91 | 0.65–1.29 | 0.601 | 0.771 |
| Asthma ever | Sugar intake | High vs low | 0.85 | 0.60–1.20 | 0.353 | 0.503 |
| Asthma ever | Soft drink intake | Moderate vs low | 1.18 | 0.90–1.53 | 0.225 | 0.369 |
| Asthma ever | Soft drink intake | High vs low | 1.06 | 0.77–1.48 | 0.714 | 0.771 |
| Hay fever ever | Sugar intake | Moderate vs low | 1.07 | 0.85–1.36 | 0.556 | 0.741 |
| Hay fever ever | Sugar intake | High vs low | 1.42 | 1.12–1.80 | 0.003 | 0.024 |
| Hay fever ever | Soft drink intake | Moderate vs low | 0.79 | 0.66–0.93 | 0.006 | 0.030 |
| Hay fever ever | Soft drink intake | High vs low | 0.71 | 0.57–0.87 | 0.001 | 0.013 |
| Eczema ever | Sugar intake | Moderate vs low | 1.29 | 0.89–1.85 | 0.175 | 0.313 |
| Eczema ever | Sugar intake | High vs low | 1.72 | 1.20–2.46 | 0.003 | 0.024 |
| Eczema ever | Soft drink intake | Moderate vs low | 0.78 | 0.62–0.99 | 0.039 | 0.129 |
| Eczema ever | Soft drink intake | High vs low | 0.71 | 0.52–0.99 | 0.042 | 0.129 |

**Supplementary Table 3:** Subgroup-specific associations for significant interaction terms. Models adjusted for BMI, soft drink intake, physical activity, traffic exposure, and sex or age where appropriate. Low sugar intake was the reference category.

| **Interaction assessed** | **Outcome** | **Subgroup** | **Sugar comparison** | **aOR** | **95% CI** | **p-value** |
| --- | --- | --- | --- | --- | --- | --- |
| Sugar intake × sex | Hay fever ever | Male | Moderate vs low | 1.66 | 1.12–2.45 | 0.012 |
| Sugar intake × sex | Hay fever ever | Male | High vs low | 2.24 | 1.52–3.32 | <0.001 |
| Sugar intake × sex | Hay fever ever | Female | Moderate vs low | 0.81 | 0.59–1.10 | 0.168 |
| Sugar intake × sex | Hay fever ever | Female | High vs low | 1.02 | 0.76–1.39 | 0.874 |
| Sugar intake × age group | Eczema ever | Younger cohort | Moderate vs low | 2.25 | 1.21–4.19 | 0.011 |
| Sugar intake × age group | Eczema ever | Younger cohort | High vs low | 2.58 | 1.40–4.75 | 0.002 |
| Sugar intake × age group | Eczema ever | Older cohort | Moderate vs low | 0.92 | 0.59–1.45 | 0.733 |
| Sugar intake × age group | Eczema ever | Older cohort | High vs low | 1.30 | 0.83–2.02 | 0.249 |

**Supplementary Table 4:** Age-group stratified sensitivity analyses for primary dietary exposure–outcome associations. Models were repeated separately in the younger parent-reported cohort aged 6–8 years and the older self-reported adolescent cohort aged 13–15 years. Models were adjusted for BMI, sugary snack intake, soft drink intake, physical activity, traffic exposure, and sex. Low intake was the reference category for both sugary snack and soft drink intake. Values are adjusted odds ratios (aORs) with 95% confidence intervals (CIs).

| **Outcome** | **Exposure** | **Comparison** | **Younger cohort, 6–8 years aOR** | **95% CI** | **Older cohort, 13–15 years aOR** | **95% CI** |
| --- | --- | --- | --- | --- | --- | --- |
| Current wheeze | Sugary snacks | Moderate vs low | 0.95 | 0.40–2.23 | 0.91 | 0.61–1.36 |
| Current wheeze | Sugary snacks | High vs low | 1.19 | 0.52–2.73 | 1.01 | 0.68–1.50 |
| Current wheeze | Soft drinks | Moderate vs low | 1.03 | 0.61–1.73 | 1.37 | 0.99–1.89 |
| Current wheeze | Soft drinks | High vs low | 0.78 | 0.18–3.40 | 1.64 | 1.16–2.33 |
| Current eczema | Sugary snacks | Moderate vs low | 2.04 | 1.02–4.11 | 0.78 | 0.55–1.09 |
| Current eczema | Sugary snacks | High vs low | 2.34 | 1.18–4.64 | 0.82 | 0.58–1.16 |
| Current eczema | Soft drinks | Moderate vs low | 0.65 | 0.43–0.96 | 0.97 | 0.74–1.28 |
| Current eczema | Soft drinks | High vs low | 0.57 | 0.20–1.68 | 1.13 | 0.83–1.54 |
| Current rhinitis | Sugary snacks | Moderate vs low | 1.05 | 0.65–1.71 | 1.21 | 0.92–1.60 |
| Current rhinitis | Sugary snacks | High vs low | 1.03 | 0.64–1.65 | 1.19 | 0.90–1.57 |
| Current rhinitis | Soft drinks | Moderate vs low | 0.63 | 0.45–0.88 | 0.81 | 0.66–1.00 |
| Current rhinitis | Soft drinks | High vs low | 0.60 | 0.24–1.48 | 0.71 | 0.55–0.90 |
| Current rhinoconjunctivitis | Sugary snacks | Moderate vs low | 1.34 | 0.58–3.10 | 1.11 | 0.78–1.59 |
| Current rhinoconjunctivitis | Sugary snacks | High vs low | 1.42 | 0.63–3.22 | 1.24 | 0.87–1.77 |
| Current rhinoconjunctivitis | Soft drinks | Moderate vs low | 0.54 | 0.30–0.97 | 0.94 | 0.72–1.22 |
| Current rhinoconjunctivitis | Soft drinks | High vs low | 1.01 | 0.29–3.43 | 0.86 | 0.63–1.16 |
| Asthma ever | Sugary snacks | Moderate vs low | 1.12 | 0.48–2.63 | 0.87 | 0.60–1.27 |
| Asthma ever | Sugary snacks | High vs low | 1.25 | 0.55–2.84 | 0.76 | 0.52–1.12 |
| Asthma ever | Soft drinks | Moderate vs low | 0.77 | 0.45–1.33 | 1.35 | 0.98–1.86 |
| Asthma ever | Soft drinks | High vs low | 1.61 | 0.54–4.81 | 1.15 | 0.80–1.66 |
| Hay fever ever | Sugary snacks | Moderate vs low | 1.18 | 0.71–1.96 | 0.99 | 0.76–1.30 |
| Hay fever ever | Sugary snacks | High vs low | 1.58 | 0.96–2.60 | 1.30 | 0.99–1.70 |
| Hay fever ever | Soft drinks | Moderate vs low | 0.64 | 0.46–0.89 | 0.90 | 0.73–1.11 |
| Hay fever ever | Soft drinks | High vs low | 0.62 | 0.26–1.46 | 0.80 | 0.63–1.01 |
| Eczema ever | Sugary snacks | Moderate vs low | 2.25 | 1.21–4.19 | 0.92 | 0.59–1.45 |
| Eczema ever | Sugary snacks | High vs low | 2.58 | 1.40–4.75 | 1.30 | 0.83–2.02 |
| Eczema ever | Soft drinks | Moderate vs low | 0.80 | 0.57–1.11 | 0.79 | 0.57–1.10 |
| Eczema ever | Soft drinks | High vs low | 0.97 | 0.43–2.22 | 0.71 | 0.48–1.04 |
| Doctor-diagnosed asthma | Sugary snacks | Moderate vs low | 1.14 | 0.49–2.68 | 0.95 | 0.64–1.42 |
| Doctor-diagnosed asthma | Sugary snacks | High vs low | 1.14 | 0.50–2.63 | 0.77 | 0.51–1.16 |
| Doctor-diagnosed asthma | Soft drinks | Moderate vs low | 0.72 | 0.41–1.28 | 1.38 | 0.98–1.92 |
| Doctor-diagnosed asthma | Soft drinks | High vs low | 2.24 | 0.81–6.17 | 1.19 | 0.81–1.74 |
| Doctor-diagnosed hay fever | Sugary snacks | Moderate vs low | 0.89 | 0.53–1.49 | 1.10 | 0.80–1.51 |
| Doctor-diagnosed hay fever | Sugary snacks | High vs low | 1.21 | 0.74–1.98 | 1.25 | 0.91–1.72 |
| Doctor-diagnosed hay fever | Soft drinks | Moderate vs low | 0.68 | 0.48–0.96 | 0.80 | 0.63–1.02 |
| Doctor-diagnosed hay fever | Soft drinks | High vs low | 0.90 | 0.40–2.07 | 0.75 | 0.57–0.98 |
| Doctor-diagnosed eczema | Sugary snacks | Moderate vs low | 1.93 | 1.01–3.70 | 0.72 | 0.41–1.26 |
| Doctor-diagnosed eczema | Sugary snacks | High vs low | 2.34 | 1.24–4.41 | 0.98 | 0.57–1.69 |
| Doctor-diagnosed eczema | Soft drinks | Moderate vs low | 0.84 | 0.59–1.19 | 0.77 | 0.50–1.19 |
| Doctor-diagnosed eczema | Soft drinks | High vs low | 0.82 | 0.33–2.05 | 0.68 | 0.41–1.13 |
